# Nano-Scale Video Imaging of Motility Machinery by High-Speed Atomic Force Microscopy

**DOI:** 10.3390/biom15020257

**Published:** 2025-02-10

**Authors:** Steven John McArthur, Kenichi Umeda, Noriyuki Kodera

**Affiliations:** 1 WPI Nano Life Science Institute (WPI-NanoLSI), Kanazawa University, Kakuma-machi, Kanazawa 920-1192, Japan; umeda.k@staff.kanazawa-u.ac.jp; 2 Precursory Research for Embryonic Science and Technology (PRESTO), Japan and Japan Science and Technology Agency (JST), 4-1-8 Honcho, Kawaguchi 332-0012, Japan

**Keywords:** motility, atomic force microscopy, high-speed AFM, cytoskeleton, molecular motor, bio-imaging, protein dynamics, supramolecular complex, single-molecule biophysics

## Abstract

Motility is a vital aspect of many forms of life, with a wide range of highly conserved as well as highly unique systems adapted to the needs of various organisms and environments. While many motility systems are well studied using structural techniques like X-ray crystallography and electron microscopy, as well as fluorescence microscopy methodologies, it is difficult to directly determine the relationship between the shape and movement of a motility system due to a notable gap in spatiotemporal resolution. Bridging this gap as well as understanding the dynamic molecular movements that underpin motility mechanisms has been challenging. The advent of high-speed atomic force microscopy (HS-AFM) has provided a new window into understanding these nano-scale machines and the dynamic processes underlying motility. In this review, we highlight some of the advances in this field, ranging from reconstituted systems and purified higher-order supramolecular complexes to live cells, in both prokaryotic and eukaryotic contexts.

## 1. Introduction

In the ~4 billion years since the emergence of life, various types of motility systems have emerged. For microorganisms, motilities such as gliding, swimming, or swarming are crucial, enabling everything from nutrient acquisition and escape from predators to the dispersal of offspring [1,2]. For multicellular organisms, motility also plays critical roles in embryonic development, tissue renewal, and the immune response [3]. To date, motility at the molecular level is categorized into 18 distinct types based on the structure of the force-producing motor [4]. According to the authors of that categorization, molecular movements that do not propel a cell or organism are not considered to be motility, so this review follows that definition. In other words, for example, molecular movements such as those seen in rotary ATPase, molecular translocation along nucleic acids, and intracellular vesicle transportation are not considered to be motility.

Understanding the dynamic processes underlying microbial and multicellular motility at the cellular and subcellular levels as well as the molecular machineries that enable them is a vital step in understanding the biology of many organisms. A wide variety of techniques have been applied, in vitro and in vivo, in an attempt to identify the structural components and physicochemical mechanisms underlying various forms of cellular motility, including fluorescence microscopy, optical tweezers, electron microscopy, and X-ray crystallography [5,6,7,8,9]. However, a common limitation is that most techniques targeting the submolecular scale can reveal only limited aspects of the mechanisms of the machineries under investigation due to their reliance on exogenous labels (e.g., fluorescence microscopy), static samples (e.g., electron microscopy), and population-averaging techniques. Label-reliant techniques only allow the visualization of labels attached to the target, while the target itself remains invisible, rendering intra-protein conformational changes very difficult to observe directly [10,11]. Conversely, label-free high-resolution techniques only provide snapshots of the targets and/or ensemble averaging, which convolutes the structures of individual molecules to obtain a consensus across the entire population [7,12,13].

Meanwhile, high-speed atomic force microscopy (HS-AFM) has established its own unique position. This is because HS-AFM has the ability to capture the structural movements and functional dynamics of biomolecules and biomolecular systems at the nanometer scale without labeling, fixing or staining the targets [14,15]. This unique ability to push the threshold of spatiotemporal resolution into a biologically relevant range has kept HS-AFM at the cutting edge of the study of many biological systems and phenomena [16,17,18,19]. Here, we will highlight some of the advances in the field of microbial and multicellular motility that HS-AFM has enabled, increasing in scale from purified and reconstituted systems to high-order supramolecular complexes, and finally to live cells.

## 2. HS-AFM Overview

**Figure 1:**
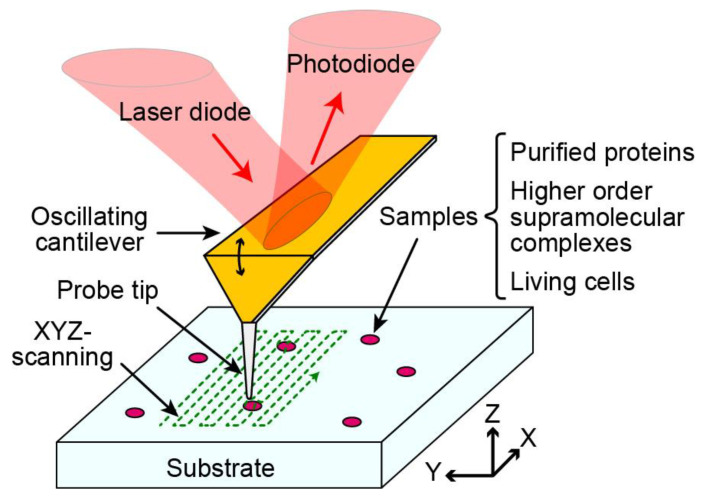
Overview of the basic mechanism of HS-AFM. The imaging principle is the same as conventional AFM. The sample of interest, which can range from highly purified proteins or higher-order supramolecular complexes to whole living cells, is bound on a substrate. This substrate is then probed in a raster pattern by a rapidly oscillating cantilever with an ultrafine tip. A laser reflected off the back of the cantilever to a detector measures the cantilever’s deflection, allowing for the mapping of height (Z) data at all positions (XY) across the scan area.

First invented in 1986, AFM is a scanning probe microscopy in which a sample is probed by an ultrafine tip mounted on the end of a microcantilever [20]. A laser reflected off the cantilever body to a positional detector measures the deflection of the cantilever as the tip interrogates the surface of the sample. While a variety of imaging modes have been described, for biological applications, this probing is typically achieved by oscillating the cantilever around its resonance frequency in the Z direction (tapping mode), with a feedback control of the cantilever’s oscillation amplitude designed to minimize the tip–sample interaction [21,22]. The output signal from the feedback control provides a measurement of the height of the sample at a single pixel with specific XY coordinates (Figure 1). The sample is typically scanned in a raster pattern over the cantilever, producing a topographical map of the sample. This map has sub-nanometer resolution in the Z direction with nanometer-scale resolution in the X and Y directions, allowing for very small and fine features to be imaged with accuracy and precision.

While early AFM experiments in biology were conducted on desiccated samples in air, the development of liquid AFM in 1989 [23], where observations were performed in a buffer at ambient or biological temperatures, has since enabled observation of biological samples, ranging in scale from single substrate-bound molecules to living cells without any labeling [24,25]. However, one of the main drawbacks of conventional liquid AFM has been its low temporal resolution, requiring 30 s or more to capture a single frame and thus precluding its use in observing subsecond-scale biological processes.

This limitation has been overcome by the advent of HS-AFM, which achieves imaging speeds of ~0.1 s per frame; this temporal resolution is within the timespan needed to capture dynamic processes and molecular motions [14,15]. Importantly, improvements to the feedback system mentioned above have ensured the consistent minimization of the force applied by the tip at such high scan speeds, ensuring that samples remain intact and unperturbed even under long-term imaging [26]. Owing to its high speed and low invasiveness, in the past 5 years alone, the power and utility of HS-AFM have been demonstrated in a wide range of biomolecular systems and processes, including cellular membranes [27,28,29,30], membrane vesicles [31,32,33,34], quaternary protein complexes and protein aggregation [35,36,37,38,39,40], individual proteins including transmembrane and peripheral membrane proteins [41,42,43,44,45,46], nucleic acids and their binding proteins [47,48,49,50], and even intrinsically disordered proteins and protein regions [51,52,53].

It is important to note that HS-AFM experiments can also be limited. While other structural techniques can image the entire structure of molecules, AFM data are generally limited to the probe-accessible upper surface of the sample, with limited ability to obtain information about the interior or ventral surface as the sample sits on the substrate. The ongoing development of computer-assisted inference of entire structures [54,55,56] and nanoendoscopy-AFM [57] has made great strides in overcoming this drawback. There is also a requirement for interaction between the sample and its substrate, typically a hard flat surface like mica or glass, or else, a supported lipid bilayer, minimizing rapid diffusion to an extent that allows stable imaging. This requirement can sometimes necessitate the fine-tuning of substrate properties via chemical functionalization [58,59].

Beyond topographical visualizations, the AFM methodology has been applied to ascertain the biophysical properties of samples; for example, using the cantilever tip to perform the nano-indentation of particles of interest [60,61,62,63,64]. Instead of rapidly oscillating the cantilever as it taps across the surface of the sample, the cantilever indents the sample at a controlled velocity, such that its deflection can be used to calculate the tip–sample force. Refinements such as dynamic force spectroscopy, whereby single protein–protein interactions are examined and quantified by functionalizing the cantilever tip with a biomolecule of interest, have been applied to achieve significant breakthroughs [65,66,67]. The coupling of force measurements with HS-AFM image acquisition to enable fast force microscopy and spectroscopy has also been described recently, with applications in the nanomechanical characterization of living systems [66,68]. Force spectroscopy has added a further dimension to the phenomena that HS-AFM can observe, helping build complete pictures of dynamic biological systems [14,67,69].

## 3. Motility Systems Characterized by HS-AFM

In examining the advances brought by HS-AFM to the field of motility, it is useful to divide the field between studies that study isolated motility system components in vitro, and those that study motility machinery in the context of entire functional supramolecular complexes in vitro as well as in vivo whole-cell imaging. To begin, we will consider prokaryotic isolated systems.

### 3.1. Isolated Prokaryotic Systems

**Figure 2:**
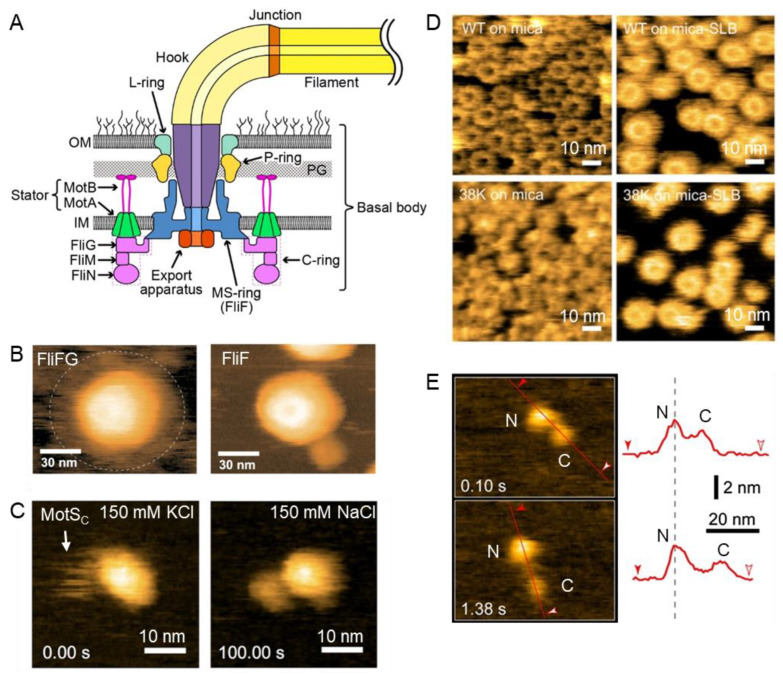
HS-AFM imaging of prokaryotic purified systems. (**A**) Schematic overview of prokaryotic flagellum. (**B**) Comparison of MS-rings composed of FliFG and FliF, with FliFG showing flexibility in the peripheral FliG region (dashed circle) [84]. (**C**) HS-AFM showing the sodium-dependent folding of MotS_C_ (white arrow) as the 150 mM KCl buffer is exchanged to 150 mM NaCl [91]. (**D**) HS-AFM demonstrating the propensity of wildtype FlhA_C_ to form rings, a property which is abnegated in the 38K mutant but can be restored by incubation on a mica-supported lipid bilayer [97]. (**E**) HS-AFM showing full-length FliK, including its N-terminal (N) and C-terminal (C) globular domains, alongside the height profiles along the red line indicated (from filled to hollow arrowheads), with profiles aligned to the peak of the N domain (dashed line) [98]; scan area: 60 × 45 nm^2^. Panels (**B**,**C**) adapted under CC-BY-NC 4.0; Panel (**D**) adapted under CC-BY-NC-SA 4.0; Panel (**E**) adapted under CC-BY-NC-ND 4.0.

One of the most important systems of prokaryotic motility is the flagellum, an elongated filament-like structure capable of differential rotary motion in response to various stimuli [1,70]. Functioning like the microscale analog of a mechanical propeller, the flagellum is composed of the following three sections: the basal body, functioning as a rotary motor including a rotor and stator; the hook, functioning as a flexible joint; and the filament, functioning as the helically moving propeller [71,72] (Figure 2A). Many different types of flagella and flagella-based motility mechanisms have been observed across the prokaryotic kingdom, including the monotrichous *Vibrio* spp. with a single polar flagellum and the peritrichous *E. coli* with multiple flagella [71,73]. HS-AFM has provided insights into the formation and functional mechanisms of the components of flagella.

The architecture of the basal body is centered on the rotor complex surrounded by up to 11 stator units, the latter of which are anchored to the peptidoglycan layer and harness the proton-motive force to provide torque to the rotor [72,74]. The rotor is composed of a transmembrane MS-ring (FliF) associated with a cytoplasmic C-ring (FliG, FliM, and FliN). Terashima and colleagues applied HS-AFM to visualize the MS-ring and the dependence of its formation on the presence and activity of FlhF/G [75], regulator proteins involved in controlling the number and position of the single flagellum in *Vibrio* and *Pseudomonas* [76,77,78,79,80,81,82,83]. Using *Vibrio* FliF that is purified and expressed in *E. coli*, which rarely assembles into the MS-ring by itself, the authors found that FlhF is required for the polar localization of FliF and directly facilitates MS-ring formation, while FlhG had no effect [75]. Another study examining the assembly of a *Vibrio* FliF-FliG fusion protein expressed in *E. coli* was performed by Takahashi and colleagues (Figure 2B) [84]. Interestingly, the structure exhibited apparent dynamic movements corresponding to FliG, seen by HS-AFM as poorly defined transitory structures around the ring. In contrast, no such mobile features were observed in the ring formed by FliF alone. This suggests that additional C-ring components, FliM and FliN, may be necessary for the formation of a stable ring structure.

The rotor complex is rotated by the torque generated by the interaction between the rotor and the stator [85,86,87]. The stator units consist of the MotAB complex, which is composed of a pentameric MotA and a dimeric MotB [86]. The proton channel activity of the stator depends on the C-terminal periplasmic domain of the B subunit, which is composed of a flexible stalk and a peptidoglycan-binding domain (PGB). This intrinsically disordered stalk is thought to act as a plug, preventing proton binding until the stator interacts with the rotor [88,89]. For *Bacillus subtilis*, in addition to the MotAB proton-dependent stator, an analogous MotPS sodium-dependent stator is also found, regulating motor association and dissociation through sodium sensing via a previously unknown mechanism [90]. Terahara and colleagues applied HS-AFM to study the MotPS complex [91]. Imaging at 0.2 s per frame enabled the visualization of two domains as follows: the C-terminal periplasmic domain of MotS (MotS_C_) and the transmembrane domain, connected by the plug-like disordered stalk. Real-time imaging while exchanging between KCl and NaCl buffers revealed that increasing sodium concentration results in the sudden folding and dimerization of the MotS_C_ PGB domain (Figure 2C). The distance between this domain and the transmembrane domain of MotPS was observed to be dynamic, suggesting that the PGB domain regulates the assembly and activation of the MotPS complex through its sodium-sensing activity and sodium-dependent folded state. These results gave rise to a new model for the mechanism and role of MotPS [91].

During the assembly of the flagellum, the basal body needs to be completed first, followed by the hook and finally the filament. Once the basal body is assembled, a type III protein export apparatus formed in the MS-ring enables the export of hook proteins. Interestingly, flagellar gene expression is tightly coupled to the assembly process, specifically at the completion of the hook–basal body complex [87]. This highly ordered protein export apparatus relies on ATPase activity as well as the proton motive force, acting as a proton/protein antiporter [92,93]. The cytoplasmic domain of FlhA (FlhA_C_) specifically forms a homononamer ring structure within this apparatus, and it plays a key role in the export and assembly of flagella [94]. FlhA_C_ consists of four domains and a flexible linker (FlhA_L_, residues 328 to 361 of FlhA_C_); filament-type substrate–chaperone complexes bind at a conserved site located at the D1/D2 interface, which leads to partial unfolding and subsequent protein translocation [95]. However, this binding site has different affinities for different substrates, suggesting an ordered filament-type protein export process [96].

Since the mechanism underlying the conformational shifts and related specificity switching in FlhA_C_ was previously poorly understood, Terahara et al. applied HS-AFM to image FlhA_C_ placed on a mica surface as well as a mica-supported lipid bilayer surface (Figure 2D) [97]. Wild-type FlhA_C_ forms a ring structure on both surfaces, while a mutant lacking most of FlhA_L_ (designated 38K) does not form this ring structure on a mica surface, suggesting that the deleted residues play a critical role in the formation of the ring. The incubation of 38K on a lipid bilayer surface restored its ability to form ring-shaped structures, albeit with a 5-fold increase in dissociation constant. Alanine mutagenesis at key residues, including E351, W354, D356, K392, and K393, which are located in various inter-monomer binding pockets, also had the effect of abrogating ring formation. These experiments demonstrate that alanine substitutions at the interface between FlhA_L_ and its neighboring subunit disrupted the ability of FlhA_L_ to form its nonameric ring structure, in turn, decreasing the affinity of FlhA_C_ for filament-type export substrates.

The hook functions as a flexible joint and its length is well controlled; in the case of *Salmonella enterica* serovar Typhimurium, the average length is 55 nm. Although the hook length was known to be maintained by the soluble protein FliK [99,100,101], structural information for full-length FliK could not be obtained using conventional methods, likely because FliK has a flexible structure. Consequently, HS-AFM experiments dedicated to visualizing its flexible structure have succeeded in clarifying the molecular shape of full-length FliK [98]. The N-terminal segment of FliK, responsible for measuring the hook length, was initially thought to be intrinsically disordered due to sequence-based prediction as well as NMR data. In contrast, HS-AFM demonstrated that a FliK molecule takes on the shape of two globules linked by a flexible string; the larger globule corresponds to the N-terminal region and the smaller one corresponds to the C-terminal region (Figure 2E). Interestingly, when the distance between the two globular domains increased, the height of the C-terminal, but not the N-terminal domain, correspondingly decreased. This suggests that, unexpectedly, the C-terminal domain unfolds more readily than the N-terminal domain; these folding tendencies were differentially affected in FliK mutants in which the hook length was not controlled. The ability of HS-AFM to directly visualize and measure the flexible FliK structure demonstrates its uniqueness among structural techniques.

### 3.2. Isolated Microtubule-Based Eukaryotic Systems

Motility in eukaryotic systems typically relies on the following two primary networks comprising the cytoskeleton: microtubules (MTs) and actin. MTs consist of hollow tubes, self-assembled from tubulin dimers [102]. Each dimer unit contains one α-tubulin and one β-tubulin molecule; these dimers polymerize head-to-tail to form protofilaments (PF), 9 to 18 (typically 13 in vivo) of which associate laterally to form the cylindrical MT [103,104]. The biological roles of MTs are numerous, including their structural roles as cytoskeletal components (including their involvement in the reinforcement of cilia and flagella) and functional roles as an intracellular motility network. Although intracellular motility is outside the scope of this review, there are insightful HS-AFM studies on the mechanical properties of MTs [105], the molecular shape of MT-associated proteins [106,107], and MT–kinesin interactions and motility [108,109,110,111].

One of the most important MT-based structures in cellular motility is the cilium, which plays a role in signal transduction (non-motile cilia) or as a mediator of cellular or extracellular fluid motility (motile cilia) [112]. The dynamic motion of motile cilia is typically rapid, undergoing a ‘beat’ at frequencies from 20 to 60 Hz [113]. This dynamic motion is underpinned by the core of the cilium, the axoneme, composed of a central pair of MTs surrounded by nine asymmetric doublet MTs (DMTs) [112]. These DMTs in turn contain a 13-PF A-tubule, attached to the 10-PF B-tubule at the inner and outer junctions [114]. Functionally, DMTs form a scaffold to which a variety of motility-related proteins and structures bind, with a highly ordered superstructure that repeats every 96 nm along their length [115]. These include axonemal dyneins, which are bound to A-tubules and slide along B-tubules to generate the main bending force of the cilium, as well as radial spokes and the nexin–dynein regulatory complex that regulates dynein activity [116,117].

**Figure 3:**
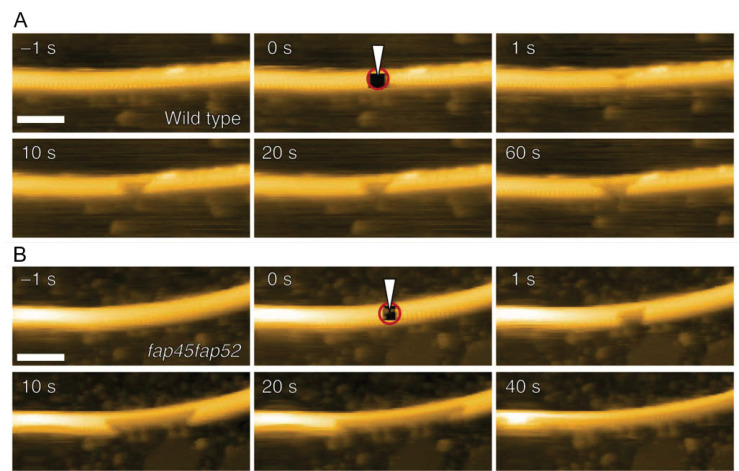
HS-AFM imaging of MT-based eukaryotic purified systems. (**A**) Upper panel, a wildtype DMT being damaged, with the hole stabilizing shortly after formation. Tip-created holes indicated by white arrowheads and red circles. (**B**) The *fap45fap52* null mutant being damaged, with extensive depolymerization following. Tip-created holes indicated by white arrowheads and red circles [124]. Figure adapted under CC-BY 4.0. Scale bar: 100 nm.

Although cytoplasmic MTs are readily damaged by bending forces [105,109,118,119,120], DMTs exhibit a highly enhanced stability that keeps them intact during their rapid beating motion. The mechanism underlying this stability has been a research focus, with microtubule inner proteins identified on the inner surfaces of A- and B-tubules, which are thought to play an important role [114,121,122,123]. Adding to this understanding, Owa and colleagues identified two previously uncharacterized flagellar associated proteins, FAP45 and FAP52, and elucidated their role through direct visualization by HS-AFM [124]. Building on the cryo-ET results that showed the destabilization of B-tubules following FAP45/52 knockout, the authors observed DMTs isolated from frayed *Chlamydomonas* axonemes on a bare mica substrate. Two DMT deposition orientations on the substrate were used for the analysis as follows: a class in which both A- and B-tubules were immobilized, exhibiting the 96 nm repeating pattern of radial spoke heads on top; and a class in which the A-tubule was immobilized with the B-tubule bound visualized directly on top, exhibiting radial spokes that extend orthogonally from the A-tubule. Defects were generated in these DMTs by the direct penetration of the cantilever [105,125], and the damage propagation from these defects, in the presence and absence of FAP45/52, was determined. As expected, in wildtype DMTs containing FAP45/52, the hole remained constant (Figure 3A), whereas in FAP45/52 knockout-derived DMTs, the hole expanded in an apolar manner (Figure 3B). The depolymerization speed was fastest in the double knockout, with single knockouts depolymerizing more slowly; however, even the FAP45/52 double knockout was more stable than control cytoplasmic MTs, implicating additional mechanical stabilization factors that remain to be investigated.

### 3.3. Isolated Actin-Based Eukaryotic Systems

Aside from MTs, the other major cytoskeletal protein component is actin, which undergoes ATP-dependent polymerization to form double-helical microfilaments [126,127]. These filaments play many vital roles in muscle contraction, amoeboid movement, cytokinesis, intracellular transport, and nuclear transcriptional regulation, among others [126,128,129,130]. All of these functions differentially depend on the spatiotemporal regulation of actin-binding proteins (ABPs); the interactions of actin with these ABPs are important fields of study that are highly amenable to HS-AFM [131,132]. While not within the scope of this review, the movement mechanisms and interactions between actin and ABPs that govern intracellular actin-based motility have been widely studied by HS-AFM. For example, vesicular transport relies on the motor protein myosin V, whose movement mechanism and actin-binding interactions have been extensively characterized by HS-AFM [133]. The mechanisms of anillin, another ABP which stabilizes the contractile ring during cell division, has been another target of HS-AFM studies [134]. Moreover, ABPs can form secondary and tertiary interactions with other proteins, which can regulate the dynamics of the ABP–actin interaction or trigger secondary effects. An example of the latter is the binding of synaptopodin 2-like protein to α-actinin, an ABP, in cardiomyocytes; this interaction was shown by HS-AFM to promote actin bundling [135].

Another ABP, cofilin, is well known for its concentration-dependent ability to either alter the structural properties of actin filaments or promote their severing [136], thereby playing a vital role in cell motility [137]. However, the regulation of cofilin has been enigmatic; for example, inactive cofilin has been shown to successfully activate cofilin-dependent processes, and constitutively active cofilin has no negative impact on actin-dependent motility [138,139]. It has been proposed that cooperative conformational changes, similar to those seen in other ABPs such as myosin, may constitute a regulatory mechanism for cofilin [140]. These conformational changes notably include a shortening of the actin filament half-helical pitch (HHP) by 25%, with sufficient potency such that a single cofilin molecule binding can trigger this change in ~100 adjacent actins [141,142,143]. In this sense, cofilin binding to actin is cooperative, in that cofilin-induced conformational changes, which promote cofilin binding, are propagated to nearby actins within the same filament [144,145]. However, technical challenges have limited our understanding of the propagation of these conformational changes.

**Figure 4:**
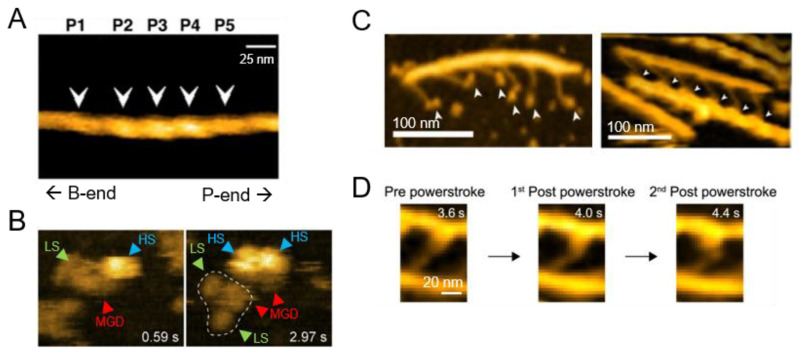
HS-AFM imaging of actin-based eukaryotic purified systems. (**A**) An actin filament with 3 bound cofilin clusters (P2–P4, white arrowheads), revealing polarity-dependent changes in actin HHP [146]. (**B**) HS-AFM observation of *Xenopus laevis* CAP-1 in complex with actin, showing the middle globular domain (MGD, red) alongside two arms that can interconvert between the low state (LS, green) and high state (HS, blue), with one complex highlighted by dashed lines [155]. Scan area: 80 × 64 nm^2^. (**C**) DNA origami-based thick filaments, both on mica (left panel) and bound to F-actin on a lipid bilayer (right panel); white arrowheads show the myosin heads [157]. (**D**) Actin-bound S1 conformational changes during powerstroke; numbers represent the acquisition times. [157]. Figure adapted under CC-BY 4.0.

In order to address this, Ngo and colleagues performed HS-AFM to better characterize cofilin-induced structural changes in actin filaments [146]. To begin, the authors observed cofilin binding in clusters along actin filaments on a mica-supported lipid bilayer substrate, with a corresponding ~2 nm increase in peak height and a concomitant 27% reduction in HHP from 36.8 nm to 26.9 nm. Interestingly, HHP alterations were found to be asymmetric when surrounding cofilin clusters, with the pointed end side of the cluster being shorter, and the barbed end side being longer (Figure 4A). The authors then used HS-AFM to visualize the growth mechanism of individual cofilin clusters, showing a domino-like increase in successive actin peaks in the filament in the direction of the pointed end. Moreover, HS-AFM observed filament severing. Notably, the cluster boundary was highly correlated with severing, with 80% of severing events occurring within one HHP length of the boundary, either inside or outside the cluster; the severing of distal bare actin regions was very rare. Altogether, HS-AFM successfully revealed the mechanism underlying cofilin cluster formation and the direction and propagation of cofilin-induced actin filament structural changes, with implications for the binding of other ABPs and filament regulation [146,147]. This understanding of cofilin–actin dynamics was recently expanded to examine the underlying mechanism by which cofilin induces structural changes in the neighboring regions of bare actin [148]. This further delineates the differences between filamentous actin (F-actin) and actin in its cofilin-binding state.

Cyclase-associated protein (CAP) is another ABP that plays an important role in the regulation of actin dynamics [149,150]. While it inhibits polymerization by directly binding actin monomers, it also facilitates nucleotide exchange, increasing the pool of actin-ATP monomers ready for polymerization [151,152]. In concert with cofilin, CAP can enhance the severing as well as dissociation of monomers from the pointed ends of actin filaments [153,154]. To clarify the native structure of the CAP–actin complex, HS-AFM of purified native *Xenopus laevis* oocyte CAP1 in complex with actin was performed [155]. The observation on a bare mica surface revealed a tripartite structure, composed of a middle globular domain flanked by two arms (Figure 4B). These arms were found to be either actin-bound (high state) or unbound (low state), with the transient binding of actin triggering the interconversion between the two. Despite a 6:6 CAP–actin stoichiometry previously observed for yeast and mouse CAP, *X. laevis* CAP1 was found to form a 4:4 complex that more closely resembles that seen in humans [156].

Another physiologically important system that relies on the activity of actin and ABPs are skeletal muscles, which contract by a sliding translocation between thick filaments composed of myosin II and thin filaments composed of actin [158]. The head (motor) and lever-arm domains are found within subfragment 1 (S1) of myosin II, while subfragment 2 (S2) contains a coiled-coil domain [159]. Myosin II motion is described by the swinging cross-bridge model, in which the swinging of the S1 lever-arm domain provides the driving force [160]. While the movement of individual non-muscle myosins on actin is well characterized [161,162], the individual dynamics of muscle myosins are difficult to observe because they are not processive in isolation but rather function in concert with other muscle myosins in the thick filaments of the sarcomere. Further insights have been difficult to obtain due to the limitations of the commonly used synthetic thick filaments, which are composed of purified self-assembled myosin II that organizes randomly rather than following the symmetric bipolar organization of the sarcomere [163].

To address this, a 3D DNA origami ‘thick filament’ was employed by Fujita and colleagues as a scaffold for recombinant human myosin II, enabling the visualization of the two-step reversible lever-arm swing and microsecond weak binding [157]. This novel system is composed of a 10-helix bundle DNA rod that is used as a backbone, with ‘linker’ 2-helix bundles spaced 42.8 nm apart to mimic myosin II S2 as binding sites for S1 via a 21-nucleotide handle. This thick filament successfully formed a rigor complex with thin actin filaments, with strong binding sites spaced 36 nm apart corresponding to actin HHP. Performing HS-AFM on thick filaments with six myosin heads each, the authors observed the successful movement along actin filaments (Figure 4C). This allowed the first observation of the muscle myosin lever-arm swing during force generation while bound to actin (Figure 4D). Consistent with the research performed by optical tweezers [164,165], a two-step powerstroke denoted by a change in the orientation of S1 relative to the actin filament was observed during translocation.

Actin-based supramolecular complexes also play important roles in mediating physiological functions such as muscle contraction [166]. In muscles, actin filaments associate with complexes of the ABPs troponin (Tn) and tropomyosin (Tm), which together comprise the thin filaments (TF) upon which myosin acts to drive contraction [167]. Examining native cardiac TFs isolated from rabbits, Matusovsky and colleagues used HS-AFM to determine that increasing Ca^2+^ concentration resulted in a significant rearrangement of the Tm strand, leading to activation [168]. The question of whether myosin II acts independently or cooperatively to bind TFs and exert force was next investigated by the authors, who found that even a single myosin head binding a cardiac TF slightly increased the probability of subsequent heads to bind, suggesting a cooperative effect [169].

### 3.4. Live Cell Imaging

Recent applications of HS-AFM have enabled the visualization of motility machinery in the most biologically relevant context of all—live cells [170,171,172,173]. Advances in this field over the last decade have enabled the direct imaging of dynamic structures at the surface of live cells—that is, outside the plasma membrane or just inside it—by employing a mix of sample-scanning and tip-scanning approaches, which have collectively deepened our understanding of motility-related phenomena [172,174,175,176].

Beyond the motility mechanisms discussed thus far, more exotic motility-related superstructures have been observed in live cells by HS-AFM. One example is the unusual method of motility employed by the parasitic *Mycoplasma mobile*. This motility mechanism is surface gliding; it appears to have arisen through a combination of repurposed cell adhesion systems and ATP synthase [177]. These cells have a defined polarity with a lightbulb shape, moving in the direction of their narrow end on surfaces [178]. The following two sets of machinery are involved in movement: the surface system and the force-generating interior system [179]. The surface system consists of the proteins Gli349, Gli521, and Gli123; Gli349 binds to sialylated oligosaccharides on the surfaces, while Gli521 and Gli123 transmit force and act as a scaffold [180,181,182,183]. The interior system consists of a massive ‘bell’ at the front apex of the cell with ~28 ‘chains’ attached, each extending along the cell axis and composed of a string of 21 × 13 nm particles [184]. The primary sequences of these particles suggest they are related to ATP synthase; indeed, they have been shown to hydrolyze ATP, implicating them as the force generator that powers movement of the surface system [179,185].

**Figure 5:**
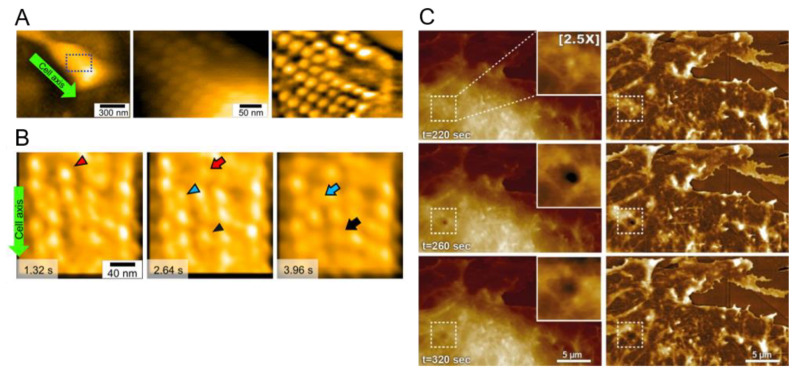
HS-AFM imaging of live cells. (**A**) An entire *M. mobile* cell immobilized on glass (left panel); higher resolution image of the outlined region showing chains of particles at the cell surface (middle panel); filtered version of the middle panel (right panel) [186]. (**B**) *M. mobile* cells imaged with 15.4 mM sodium azide, showing three consecutive frames. Movements of three selected particles are shown by red, blue, and black arrowheads (initial position) and arrows (final position), demonstrating movement to the left relative to the cell axis [186]. (**C**) Live-cell HS-AFM imaging of a fibroblast. Right panels are processed versions of left panels, highlighting the cortical cytoskeleton. Dashed boxes and insets highlight a transient invagination resembling an endocytosis event [188]. Figure adapted under CC-BY 4.0.

Since the dynamic behavior and nature of movements in this internal system had yet to be characterized, Kobayashi and colleagues performed live-cell HS-AFM [186]. HS-AFM on live cells revealed large-scale particle matrices at the front of the cells, strung roughly along the cell axis at a pitch of ~31.5 nm (Figure 5A). Individual particles were about 2 nm in height and measured 27 × 14 nm laterally, with height profiles showing two peaks 10 nm apart within each particle. Tracing individual particle displacements in the presence of sodium azide to reduce ATPase activity and therefore cell gliding speed, most particles were observed to translocate ~9 nm to the left (relative to the cell axis) and ~2.3 nm inwards over ~330 ms (Figure 5B); some particles showed an equivalent returning movement as well. Thus, the dynamic behavior and structure of the interior gliding machinery were visualized for the first time [186]. A subsequent study using both cryo-EM and HS-AFM on purified particles successfully elucidated their structure, which was found to be composed of a dimer of rotary F_1_-like ATPase units arranged in a 17-dimer chain [187].

Live-cell HS-AFM has also been applied to the observation of mammalian cells. By combining HS-AFM with fluorescence microscopy, Yoshida and colleagues succeeded in capturing real-time membrane invagination events resembling endocytosis/exocytosis as well as their corresponding cortical actin network dynamics; the authors even observed the cytosolic movement of mitochondria [173]. Ongoing technical improvements have furthered the usage of HS-AFM in live cell applications. For example, a recent paper by Marchesi and colleagues describes a wide-range scanner, featuring a 36 × 36 µm^2^ scan area with ~4 nm resolution [188]. This scanner represents a step forward from the 15 µm and 23 µm scanners described previously, which were limited to pixel sizes of approximately 78 nm [172,189]. Scanning a 27 × 16 µm^2^ region at the edge of a fibroblast at a nominal resolution of 17 × 17 nm, the authors were able to observe the ongoing reorganization of the cortical cytoskeleton (Figure 5C). In addition, a transient depression with a lifetime of around 80 s was observed, suggestive of an endocytotic event. This novel scanner thus allows for the live-cell HS-AFM imaging of scan areas not previously achievable, and it enables dynamic observations from molecular- to submolecular-level resolutions within that area. Complementary improvements in image analysis, including a recently described machine learning technique for quantitatively reconstructing actin filaments from live-cell HS-AFM images, further highlight the promise of this burgeoning methodology [190].

## 4. Conclusions and Perspectives

From single molecules to complex supramolecular systems and even live cells, the advent of HS-AFM has opened an ever-expanding window into the nano-scale biological world. The direct visualization of motility machineries in action by HS-AFM provides the basis for a straightforward understanding of how these machineries are constructed and how they perform their functions. As the frontiers of spatiotemporal resolution are pushed further to allow for the imaging of finer and faster details [19,58,191,192,193,194], the variety of biomolecular processes that can be directly visualized is expected to increase. In addition, computer-assisted HS-AFM approaches that tackle existing spatiotemporal resolution limits and other drawbacks associated with probe microscopy are also expected to further provide deep mechanistic insight into motility machinery systems and microorganisms [19,195,196,197,198].

Although 18 types of motility machinery have been categorized so far [4], unrecognized types of motility machinery may exist in organisms that are difficult or impossible to culture, such as the Candidate Phyla Radiation subgroup of bacteria [4,199]. We are hopeful that, upon the future discovery of novel motility machinery, HS-AFM will be a key method and frontline tool for scientists to observe and understand its structural dynamics and functional mechanism. We eagerly anticipate the next generation of breakthroughs in the field of cellular motility.

## Data Availability

Not applicable.
